# Hepatocellular carcinoma-targeting oncolytic adenovirus overcomes hypoxic tumor microenvironment and effectively disperses through both central and peripheral tumor regions

**DOI:** 10.1038/s41598-018-20268-6

**Published:** 2018-02-02

**Authors:** A-Rum Yoon, JinWoo Hong, Minjung Kim, Chae-Ok Yun

**Affiliations:** Department of Bioengineering, College of Engineering, Hanyang University, 222 Wangsimni-ro, Seongdong-gu, Seoul 04763 Korea

## Abstract

Cancer-specific promoter driven replication of oncolytic adenovirus (Ad) is cancer-specific, but shows low transcriptional activity. Thus, we generated several chimeric α-fetoprotein (AFP) promoter variants, containing reconstituted enhancer and silencer regions, to preferentially drive Ad replication in hepatocellular carcinoma (HCC). Modified AFP promoter, containing 2 enhancer A regions and a single enhancer B region (a2bm), showed strong and HCC-specific transcription. In AFP-positive HCCs, gene expression was 43- to 456-fold higher than those of control AFP promoter lacking enhancers. a2bm promoter was further modified by inserting multiple hypoxia-responsive elements (HRE) to generate Ha2bm promoter, which showed stronger transcriptional activity than a2bm promoter under hypoxic conditions. Ha2bm promoter-regulated oncolytic Ad (Ha2bm-d19) showed a stronger antitumor and proapoptotic effect than did a2bm promoter-regulated oncolytic Ad (a2bm-d19) in HCC xenograft tumors. Systemically administered Ha2bm-d19 caused no observable hepatotoxicity, whereas control replication-competent Ad, lacking cancer specificity (d19), induced significant hepatic damage. Ha2bm-d19 caused significantly lower expression of interleukin-6 than d19, showing that HCC-targeted delivery of Ad attenuates induction of the innate immune response against Ad. This chimeric AFP promoter enabled Ad to overcome the hypoxic tumor microenvironment and target HCC with high specificity, rendering it a promising candidate for the treatment of aggressive HCCs.

## Introduction

Liver cancer is the tenth most common malignant tumor worldwide and accounts for a large fraction of overall cancer mortality^1^. The most common type of liver cancer, hepatocellular carcinoma (HCC), currently lacks effective treatment options^2–4^. Oncolytic adenovirus (Ad) is emerging as a promising new modality for cancer treatment because it can selectively replicate and produce its progenies in cancer cells, inducing tumor cell lysis; this is a function that no anti-cancer drugs can mimic^5,6^. Several strategies, such as partial deletion of the viral genome^7–9^ or placing the Ad E1A gene, which is essential for viral replication, under the control of tumor-specific promoters, can be used to provide Ad with tumor specificity^10,11^. These strategies culminated into commercialization of H101, which is an Ad E1B 55 kDa gene-deleted oncolytic Ad^12,13^. However, the deletion of Ad E1B 55 kDa, which endows Ad with cancer specificity, attenuates the viral production and antitumor efficacy of oncolytic Ads; thus, using H101 as a monotherapy provides insufficient clinical benefits. Ultimately, the development of an oncolytic Ad, with a high level of lytic activity and good tumor specificity, remains a critical challenge^14^.

Recent approaches mainly focus on utilizing the cancer-specific promoters to express viral genes required for viral replication^10,11^. Among several tissue-specific promoters, the α-fetoprotein (AFP) promoter is a promising candidate for providing oncolytic Ads with specificity toward HCC; this is because AFP is preferentially overexpressed in 70% of patients with liver cancer^15^. AFP promoter-driven transgene expression is highly HCC-specific^16^. However, one drawback of these cancer-specific promoters is their limited transcriptional activity, which is lower than that of conventional promoters, such as that of cytomegalovirus (CMV) promoter, composite CAG promoter (consisting of the CMV immediate early enhancer and the chicken beta-actin promoter),or elongation factor 1 alpha promoter^17–19^. Indeed, the AFP promoter has shown 500-fold less activity than the CAG promoter, limiting the utility of the AFP promoter^19^. Because insufficient gene expression by vectors remains a critical hurdle in clinical trials, these limitations severely restrict the transition of tissue-specific promoter-driven oncolytic viruses into the clinic.

To enhance the transcriptional activity of cancer-specific promoters, various modification strategies, such as addition, deletion, or chimerization of promoter domains, and exploiting the constitutively active cancer signaling pathways and transcription factors overexpressed in the tumor microenvironments, have been explored^20–23^. Kim *et al*. have demonstrated that insertion of additional c-Myc and Sp1 binding sites into the human telomerase reverse transcriptase (hTERT) promoter significantly enhances the transcriptional activity while retaining its cancer specificity^10^. Importantly, an oncolytic Ad, replicating under the control of the modified hTERT promoter (Ad-mTERT-Δ19), showed more viral replication, cytopathic effect, and antitumor efficacy than the wild type hTERT-driven oncolytic Ad^10^. Similarly, the chimeric enhancer pART, which possesses four tandemly repeated T-cell factor/lymphoid enhancer factor response elements of cyclooxygenase-2 upstream of the osteocalcin nucleosome sequence signal, significantly enhances the transcriptional activity of human regenerating islet-derived 1 alpha/pancreatic stone protein/pancreatic thread protein gene^20^.

For the HCC-specific AFP promoter, there are two types of enhancer domains existing in a region far upstream (−3.8 and −3.5 kb) of the AFP gene^24,25^. Further, two types of silencers, which are critical for regulating promoter activity and maintaining specificity of the AFP promoter, are located between the enhancer region and HCC-specific promoter region. These regulatory sequences are key regulators of AFP promoter activity. Based on this rationale, we hypothesized that reconstitution and chimerization of regions upstream of the AFP promoter, with different combinations of endogenous AFP enhancers, silencer domains, and hypoxia-response elements (HREs), may augment the transcriptional activity of the AFP promoter. Our findings demonstrate that hypoxia-responsive and enhancer region-modified AFP promoter can efficiently drive the replication of oncolytic Ad in a highly HCC-specific manner and enable the virus to overcome the hypoxic tumor microenvironment, ultimately resulting in potent inhibition of tumor growth with minimal hepatotoxicity.

## Results

### HCC-specific and strong promoter activity of modified AFP promoter variants

**Figure 1:**
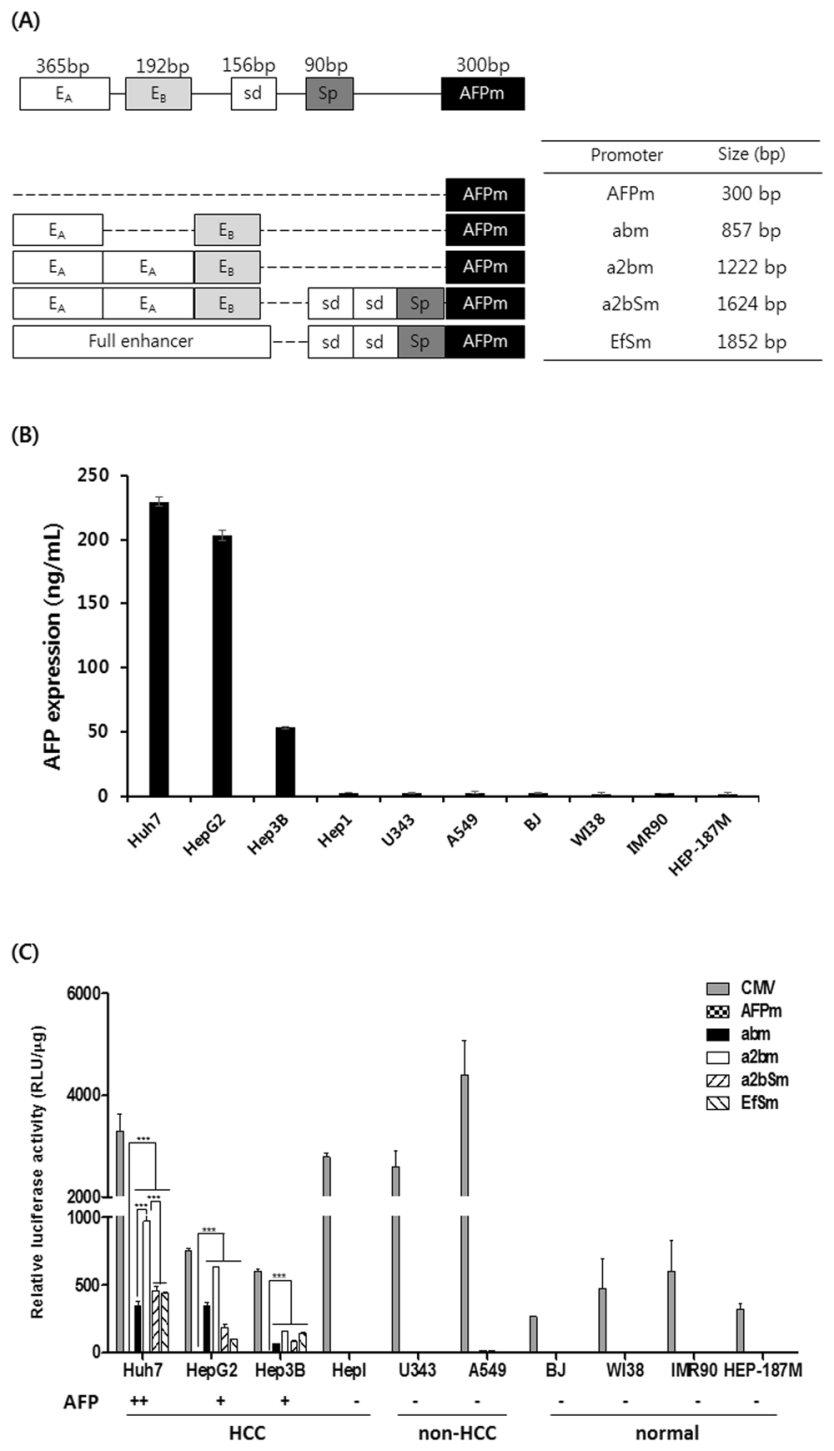
Construction and evaluation of AFP promoters. (**A**) Structural diagram of the upstream region of the α-fetoprotein (AFP) promoter variants. (**B**) Quantitative ELISA demonstrating AFP expression level in different types of cells. AFP secretion levels were determined with conventional ELISA kit using the culture supernatant harvested at 48 h after cell seeding. Each data point indicates means ± SD. (**C**) Relative levels of luciferase expression mediated by CMV or different AFP promoter variants with respect to the AFPm promoter-mediated expression of luciferase in hepatocellular carcinoma (HCC) cells, non-HCC cancer cells, and normal cells. Each cell line was tested at least three times and the data shown are representative of experiments performed in triplicate. Bars represent mean ± SD.

The expression of Ad E1A driven by the full-length AFP promoter results in HCC-specific replication of oncolytic Ad; however, it exhibits relatively low transcriptional activity and insufficient antitumor efficacy^16^. To enhance the transcriptional activity of the AFP promoter, we generated several variants of AFP promoters containing different combinations of enhancer and silencer domains located upstream of the promoter. We designed four different kinds of vectors, containing varying copy numbers of enhancer A (E_A_; 365 bp), enhancer B (E_B_; 192 bp), distal silencer (S_d_; 156 bp), proximal silencer (S_p_; 90 bp), or full enhancer segment (Ef; 1150 bp), located upstream of the minimal AFP promoter (AFPm; 300 bp),and generated promoters abm, a2bm, a2bSm, and EfSm (Fig. 1A).

To assess HCC specificity and transcriptional activity of the AFP promoter variants, we constructed pGL3 basic vectors expressing the luciferase gene under the control of the different promoter variants. First, AFP expression level was assessed in various types of cells and categorized into AFP-positive HCC cells (Huh7, HepG2, and Hep3B), AFP-negative HCC cells (HepI), non-HCC cancer cells (U343 and A549), normal fibroblasts (BJ, WI38, and IMR90), and primary human hepatocytes (HEP-187M) (Fig. 1B). These cells were subsequently transfected with pGL3/CMV, pGL3/AFPm, pGL3/abm, pGL3/a2bm, pGL3/a2bSm, or pGL3/EfSm. As shown in Fig. 1C, luciferase activity controlled by CMV promoter was the highest in all cell lines (both cancerous and normal) regardless of cellular AFP expression level, showing that CMV is potent promoter that lacks cancer specificity. All AFP promoter variants selectively induced the expression of luciferase in AFP-positive HCC cells, whereas minimal to no luciferase activity was observed in AFP-negative cancer cells and normal cells, indicating that the newly generated AFP promoter variants expressed target genes selectively in AFP-positive HCCs. Furthermore, all the enhancer and/or silencer containing AFP promoter variants induced significantly higher expression of luciferase in AFP-positive HCC cells than did the control AFPm promoter-driven plasmid lacking the enhancer and silencer domains (66- to 972-fold increase; *P* < 0.001). Of these modified AFP promoter variants, the a2bm promoter, containing two E_A_ domains and lacking the silencer domains, showed the greatest luciferase activity in AFP-positive HCC cell lines. Specifically, the a2bm promoter showed 2.1-, 2.2-, or 2.8-fold higher luciferase activity compared with that of a2bSm, EfSm, or abm promoter in AFP-positive Huh7 cells, respectively (*P* < 0.001). Notably, the specificity of the a2bm promoter was similar to those of the a2bSm promoter, showing that removal of the silencer regions did not affect the specificity of the AFP promoter variants. Based on these results, the a2bm promoter and its control promoter a2bSm were utilized in subsequent studies.

### Efficient transgene expression mediated by replication-incompetent Ad under the control of hypoxia-responsive and enhancer region-modified AFP promoter

**Figure 2:**
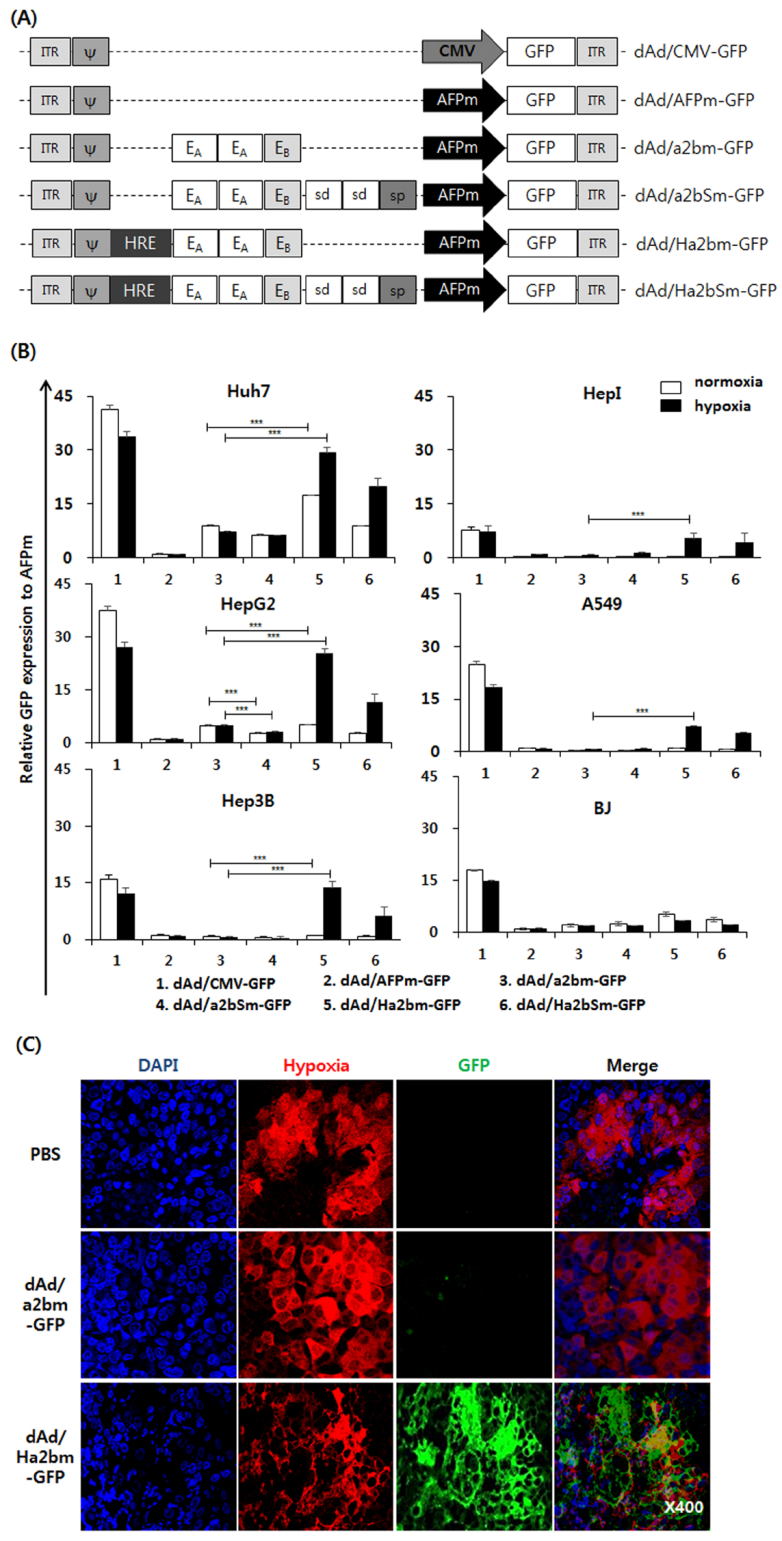
Construction and characterization of replication-incompetent Ads expressing GFP under the control of chimeric AFP promoters. (**A**) dAd/CMV-GFP Schematics of replication-incompetent Ads expressing the green fluorescent protein (GFP) under the control of various chimeric AFP promoters. (**B**) Quantification of the relative expression of GFP mediated by each viral vector. Cells were transduced with dAd/CMV-GFP, dAd/AFPm-GFP, dAd/a2bm-GFP, dAd/a2bSm-GFP, dAd/Ha2bm-GFP, or dAd/Ha2bSm-GFP. The expression levels of GFP were analyzed at 48 h post transduction by fluorescence-activated cell sorting (FACS) analysis. The expression of GFP was normalized to the expression of GFP mediated by dAd/AFPm-GFP. Each cell line was tested at least three times and the data shown are representative of experiments performed in triplicate. Bars represent mean ± SD. (**C**) Subcutaneously established Hep3B tumors were treated with 5 × 10^9^ VP of either dAd/a2bm-GFP or dAd/Ha2bm-GFP, and the control was treated with phosphate buffered saline (PBS), by intratumoral injection every 2 days for a total of three times. At 7 days post final injection, tumor tissues were treated with pimonidazole. Tissues were stained for the detection of the hypoxic region (red), anti-GFP Ab (green), and counterstained with 4′,6-diamidino-2-phenylindole (DAPI) (blue). All data are presented at x400 magnification.

Hypoxia, a hallmark of solid tumors, renders tumor cells more resistant to standard cancer therapies and promotes tumor growth^26–29^. Importantly, hypoxia attenuates the viral replication of oncolytic Ads, ultimately lessening their antitumor efficacy^30,31^. To generate a promoter candidate that can drive HCC-specific replication of oncolytic Ads and overcome the hypoxia-mediated downregulation of viral replication, a2bm and a2bSm promoters, containing six copies of HREs in the upstream region, were generated to drive the expression of GFP mediated by a replication-incompetent Ad (dAd/Ha2bm-GFP and dAd/Ha2bSm-GFP; Fig. 2A). Ads expressing GFP under the control of AFPm, a2bm, and a2bSm promoters lacking HRE domains were utilized as controls (dAd/AFPm-GFP, dAd/a2bm-GFP, and dAd/a2bSm-GFP, respectively). Replication-incompetent Ad expressing GFP under control of CMV promoter was utilized as a positive control. As shown in Fig. 2B, the expression of GFP, driven by all of the modified AFP promoter variants, was preferentially induced in AFP-positive HCC cells (Huh7, HepG2, and Hep3B), whereas it was minimally detected in AFP-negative cells (HepI, A549, and BJ) under normoxic conditions. This demonstrates that all the modifications to the upstream region of the AFPm promoter did not affect AFP specificity of the promoter. Furthermore, promoter variants without silencer domains (a2bm and Ha2bm) demonstrated a greater expression of GFP than did the variants containing the silencer (a2bSm and Ha2bSm) under normoxia and hypoxia. In HepG2 cells, a2bm exhibited 1.7- and 1.6-fold greater expression of GFP than did a2bSm under normoxic and hypoxic conditions, respectively (*P* < 0.01). Similar enhancement of transcriptional activity was observed in other AFP-positive cells (Huh7 and Hep3B) treated with dAd/Ha2bm-GFP, demonstrating that deletion of the endogenous silencer domains can greatly augment the transcriptional activity of the AFP promoter without harming the promoter’s specificity toward AFP-positive HCC.

Hypoxia-responsive Ha2bm and Ha2bSm promoters induced a markedly higher expression of GFP than did their counterpart a2bm and a2bSm in AFP-positive HCCs under normoxic and hypoxic conditions. In Huh7 cells, Ha2bm exhibited 2.0- and 4.0-fold higher expression of GFP than did a2bm under normoxia and hypoxia, respectively (*P* < 0.001). Similar trends were observed in other AFP-positive HCCs (HepG2 and Hep3B). Of note, dAd/Ha2bm-GFP vector induced comparable GFP expression level as those transduced with dAd/CMV-GFP in AFP-positive HCCs under hypoxic condition; this was achieved by transcriptional activity of dAd/CMV-GFP being downregulated under hypoxic conditions while that of dAd/Ha2bm-GFP was significantly upregulated, thus showing that hypoxia-responsiveness of promoter is essential to retain high level of gene expression via Ad vector under hypoxic conditions emulating those of solid tumors. Interestingly, the Ha2bm promoter augmented the expression of GFP in AFP-negative HCC and non-HCC cancer cells while being inactive in normal cells under hypoxic conditions. These results show that insertion of HREs into the upstream region of the AFP promoter can greatly augment cancer-specific transcriptional activity under normoxic and hypoxic conditions.

Furthermore, immunofluorescence staining of Hep3B tumors, injected with dAd/Ha_2_bm-GFP, showed a markedly greater expression of GFP than did dAd/a2bm-GFP-treated tumors in hypoxic tumor regions (Fig. 2C); this suggests that Ha2bmis highly effective for targeting hypoxic HCC tumors and overcoming the hypoxia-mediated downregulation of Ad gene expression. Taken together, these results indicate that the hypoxia-responsive Ha2bm promoter can enable Ads to effectively replicate in the hypoxic tumor microenvironment of both AFP-positive and -negative cancer.

### Potent cancer cell killing efficacy of oncolytic Ad controlled by hypoxia-responsive and enhancer region-modified AFP promoter

**Figure 3:**
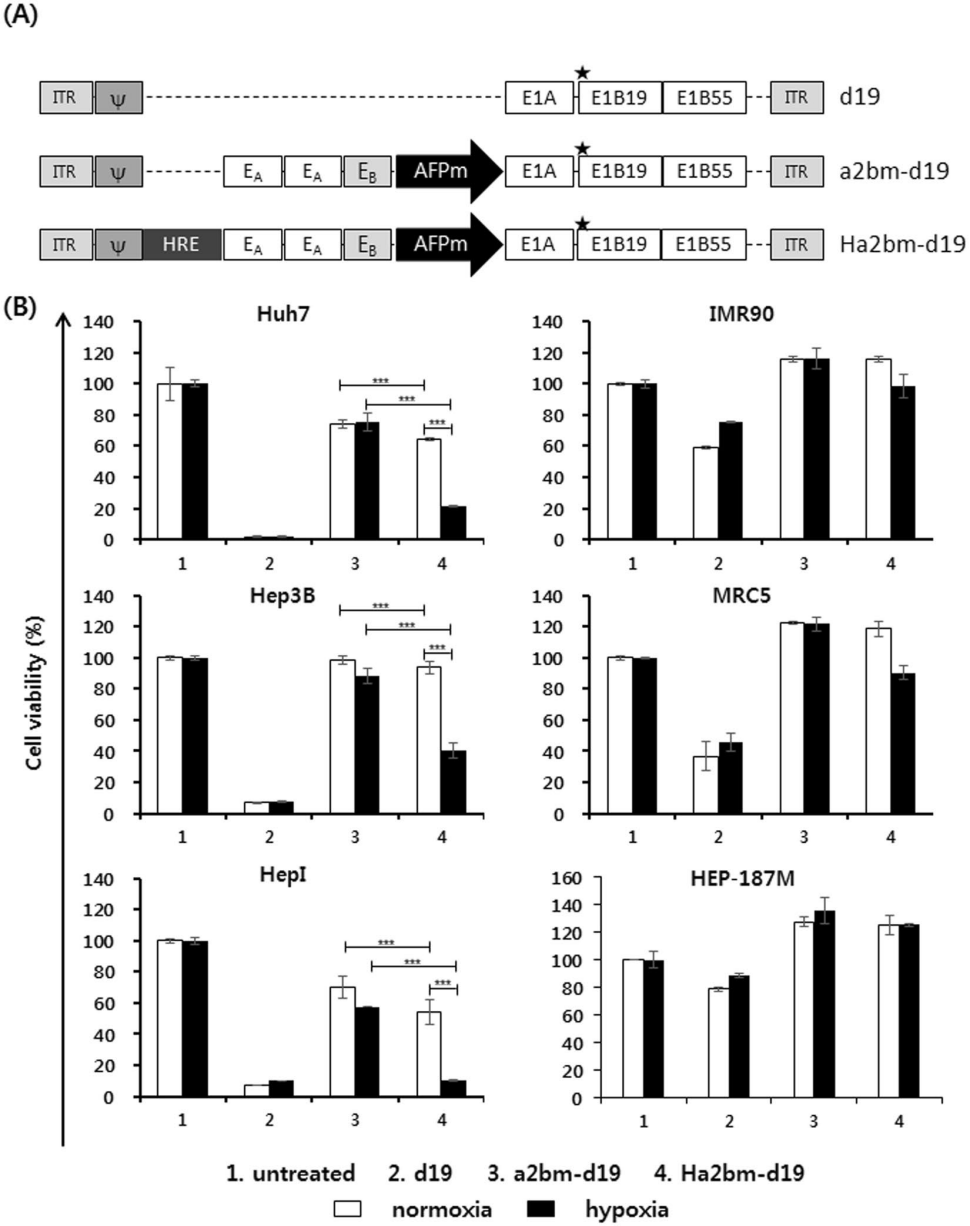
Construction and characterization of HCC-targeting oncolytic Ad regulated by chimeric AFP promoters. (**A**) Schematics of HCC-specific oncolytic Ads utilized in this study. Star stands for the stop codon replacing the start codon of the E1B 19 kDa gene. (**B**) The cell killing effect of oncolytic Ads. Cells were infected with d19, a2bm-d19, or Ha2bm-d19. At 3 to 5 days post infection, the supernatant was discarded and a 3-(4,5-dimethylthiazol-2-yl)-2,5-diphenyltetrazolium bromide (MTT) assay was performed. Each cell line was tested at least three times and the data shown are representative of experiments performed in triplicate. Bars represent mean ± SD.

Because the modified AFP promoter, in conjunction with HREs, could induce a high level of gene expression by replication-incompetent Ads in a HCC-specific manner, we constructed a series of oncolytic Ads, incorporating these promoter variants, to effectively target AFP-positive HCCs. An oncolytic Ad, which does not transcribe the E1B 19 kDa gene (d19)^14^, can induce a potent cell killing effect but lacks cancer specificity. Thus, the d19 backbone was chosen to generate an HCC-specific oncolytic Ad by regulating the expression of E1A with either an enhancer region-modified AFP promoter (a2bm) or hypoxia-responsive variant (Ha2bm) promoter, producing a2bm-d19 and Ha2bm-d19, respectively (Fig. 3A).

To assess the HCC specificity and cancer cell killing efficacy of the newly generated oncolytic Ads, HCC cell lines (Huh7, Hep3B, and HepI), normal fibroblast cell lines (IMR90 and MRC5), and primary human hepatocyte (HEP-187M) were infected with d19, a2bm-d19, or Ha2bm-d19. In agreement with a previous report, d19 induced potent cell killing activity in cancerous and normal cells (Fig. 3B), confirming that d19 lacks any cancer selectivity^14^. Conversely, a2bm-d19 and Ha2bm-d19 preferentially induced the cell killing effect in AFP-positive HCC cells while exhibiting minimal to no toxicity in normal cells and primary human hepatocytes under normoxic and hypoxic conditions. Importantly, Ha2bm-d19 showed a more potent cell killing effect than did a2bm-d19 in all the HCC cells under normoxic and hypoxic conditions (*P* < 0.001). Under normoxia, Ha2bm-d19 showed 9.8% more cytotoxicity than a2bm-d19 in AFP-positive HCC cells (Huh7). Furthermore, the enhancer function of HRE was more evident under hypoxic conditions, with Ha2bm-d19 exhibiting a 71.8% greater cancer cell killing effect than that of a2bm-d19 (*P* < 0.001). Interestingly, the cancer cell killing activity of Ha2bm-d19 was increased by 54.4% under hypoxia, as compared to under normoxia, in AFP-negative HCC cells (HepI; *P* < 0.001), indicating that HREs can enable oncolytic Ads to target the hypoxic tumor microenvironment of HCC. Taken together, these results demonstrate that the oncolytic Ad, driven by the hypoxia-responsive and enhancer region-modified AFP promoter, can induce a potent oncolytic effect in a highly HCC-specific manner under hypoxic conditions.

### Potent antitumor effect of hypoxia-responsive oncolytic Ad driven by enhancer region-modified AFP promoter, in the HCC xenograft model

**Figure 4:**
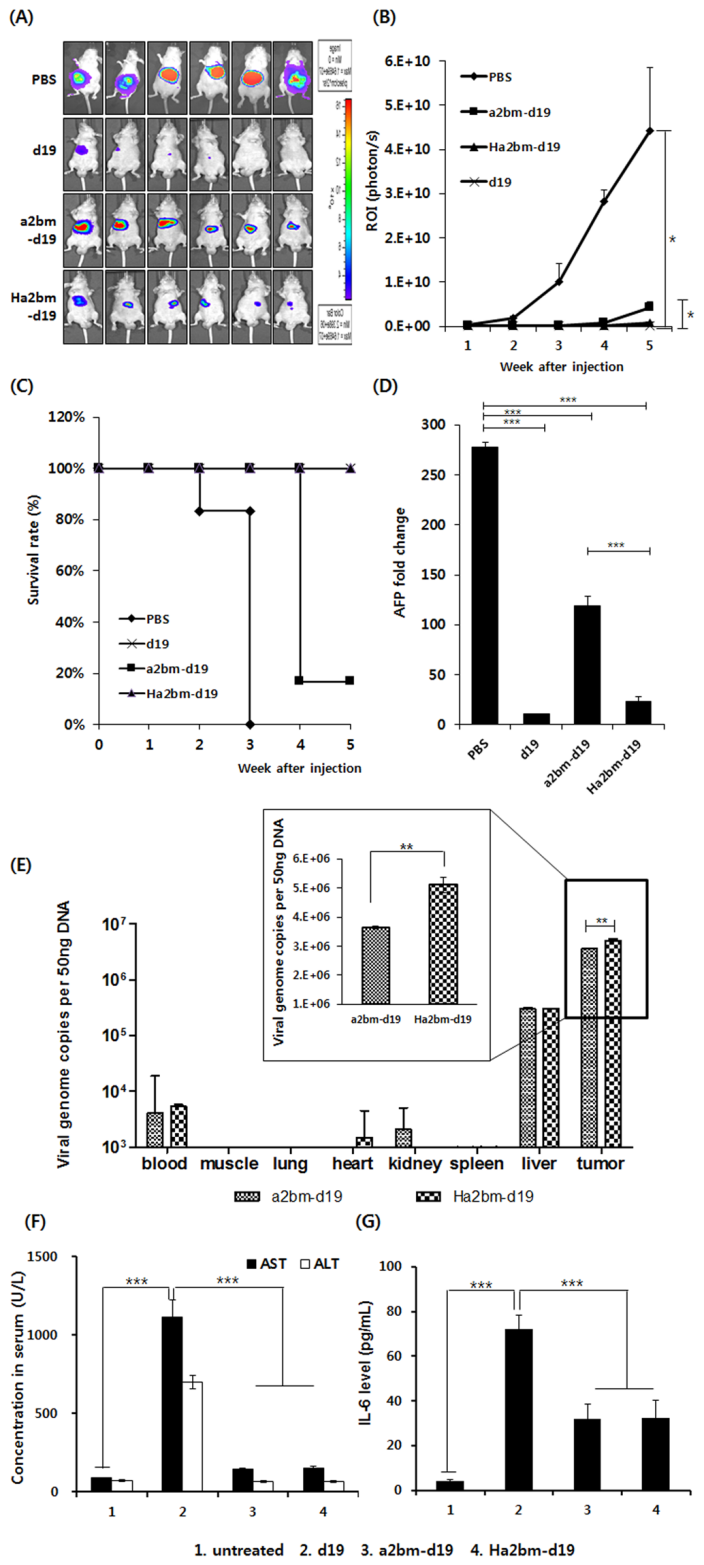
Antitumor effect of HCC-specific oncolytic Ad in orthotopic HCC model. (**A**,**B**) Luciferase-expressing Hep3B cells were injected directly into the left lobe of the liver in mice. Once the expression of AFP reached 300 ng/mL, PBS or 2.5 × 10^10^ VP of d19, a2bm-d19, or Ha2bm-d19 was intravenously injected every 2 days for a total of three times. Tumor growth was analyzed every week by optical imaging. *P < 0.05. (**C**) The percentage of surviving mice was determined by monitoring tumor growth-related events (ROI (p/s) > 2 × 10^9^ for orthotopic tumor) over the given time periods. ***P < 0.001. ***P < 0.001. (**D**) Fold changes in the serum levels of AFP in each treatment group, starting from week 0 (before treatment) to week 4 of treatment were assessed by ELISA. ***P < 0.001. (**E**) PBS, a2bm-d19, or Ha2bm-d19 was systemically injected total of 3 times every other day into the tail vein of the mice (n = 3 per group). The blood, muscle, lung, heart, kidney, spleen, liver, and tumor tissues were harvested at 72 h after the third injection. Real-time quantitative PCR was performed to detect Ad genomes. Data was normalized by subtracting values from PBS-treated group and presented as mean ± SD. **P < 0.01. (**F**) Serum ALT and AST levels were measured on day 3 after the systemic administration of PBS, d19, a2bm-d19, or Ha2bm-d19. Data represent mean ± SD. ***P < 0.001. (**G**) PBS, d19, a2bm-d19, or Ha2bm-d19 was systemically injected into mice. At 6 h post injection, serum was collected, and IL-6 levels were quantified by ELISA. Bars represent mean ± SD. ***P < 0.001.

Orthotropic tumor models are important in cancer research because of their clinical relevance^32^. To evaluate the antitumor effect of oncolytic Ads, Hep3B orthotopic xenograft tumors were established in nu/nu mice and then treated with PBS, d19, a2bm-d19, or Ha2bm-d19 via intravenous injection (3 injections of 2.5 × 10^10^ VP). PBS-treated orthotopic Hep3B tumors grew rapidly, and the luciferase signal increased to an average of 4.2 × 10^10^ ± 1.4 × 10^10^ p/s by week 5 following the initial treatment (Fig. 4A and B). In marked contrast, d19-, a2bm-d19-, or Ha2bm-d19-treated tumors reached an average total flux of 4.6 × 10^7^ ± 1.9 × 10^7^ p/s, 4.3 × 10^9^ ± 1.2 × 10^9^ p/s, and 7.8 × 10^8^ ± 1.9 × 10^8^ p/s, showing 99.9%, 90.2%, and 98.2% growth inhibition, respectively, compared with the PBS-treated tumors (*P* < 0.05; PBS versus d19, a2bm-d19 or Ha2bm-d19). Importantly, Ha2bm-d19-treated tumors showed 82% inhibition of tumor growth compared with that of a2bm-d19-treated tumors (*P* < 0.05). In addition, survival rates were also significantly higher in mice treated with d19 or Ha2bm-d19 compared with a2bm-19-treated mice in Hep3B orthotopic model (*P* < 0.001; Fig. 4C). To further evaluate the therapeutic efficacy of oncolytic Ads against HCC, serum levels of AFP, which is a good marker for gauging the malignancy of HCC and disease progression^33^, were analyzed at the start of treatment and 5 weeks after the initial treatment. As shown in Fig. 4D, a strong positive correlation between tumor growth and elevated levels of AFP was observed. The level of AFP in the serum was meaningfully elevated in PBS-treated mice (235-fold higher than those observed at baseline); d19, a2bm-d19, and Ha2bm-d19-treated groups showed AFP levels that were suppressed to 96.2%, 80.0%, and 91.5% compared with those of PBS-treated controls, respectively, at 5 weeks post treatment (*P* < 0.001). These results demonstrate that Ha2bm promoter-driven oncolytic Ad can induce potent antitumor activity against AFP-positive HCC tumors.

Two of the critical hurdles of Ad-mediated cancer gene therapy are the undesirable liver tropism and immunogenicity of Ad, which can induce hepatotoxicity and adverse inflammatory response, respectively^11,31^. Thus, we next assessed whether oncolytic Ads can selectively kill HCC tumors without damaging the normal liver tissues and avoiding the induction of adverse inflammatory immune response. As shown in Fig. 4E, highest quantity of virions (5.2 × 10^6^ viral copies) was detected in tumor tissues following systemic administration of Ha2bm-d19 at 3 days post final injection. Of note, a2bm-d19 and Ha2bm-d19 was detected at 11.1- and 15.5-fold lower quantity in normal hepatic tissues in comparison to HCC tumor tissues, respectively, suggesting that minimal quantity of virus was replicated in the normal tissues. Of consideration, the difference in hepatic and intratumoral accumulation of Ads may be overrepresented in current animal model system due to human Ad replicating poorly in murine hepatocytes while it can replicate more efficiently in human xenograft tumors^34–36^. Additionally, the native hepatic tropism of Ad leads to preferential sequestration of systemically administered Ad to both normal hepatic tissues and orthotopically implanted HCC tumor tissues via hexon-FX-HSPG interaction^34–36^. However, it should be noted that tightly regulated viral replication of AFP promoter-regulated oncolytic Ads (Ha2bm-d19 and a2bm-d19) in HCC could lead to significantly higher accumulation of these viruses in tumors than normal liver tissues. In line with these results, a2bm-d19- and Ha2bm-d19-treated mice showed no observable hepatotoxicity with serum AST/ALT level being similar to those of untreated mice, suggesting that minute quantity of virion delivered to normal hepatic tissue did not replicate and induce adverse cytopathic effect (Fig. 4F). In marked contrast, the systemic administration of the d19 Ad vector, which does not have cancer specificity, led to the significant elevation of serum AST/ALT levels, indicating that extensive hepatic damage had occurred. Similar results were observed with pro-inflammatory marker IL-6 where both oncolytic Ads, replicating under the control of AFP-specific promoters (a2bm-d19 and Ha2bm-d19), induced a significantly lower expression of IL-6 than did d19, suggesting that HCC-specific replication of oncolytic Ads may attenuate induction of the innate immune response against Ad (Fig. 4G; *P* < 0.001 versus d19). Together, these results suggest that HCC-specific oncolytic Ads can induce a potent antitumor effect against HCC with high specificity and elicit minimal cytopathic effect in adjacent normal tissues.

### Extensive viral distribution and potent induction of apoptosis by hypoxia-responsive oncolytic Ad, driven by the enhancer region-modified AFP promoter, in HCC tumor xenografts

**Figure 5:**
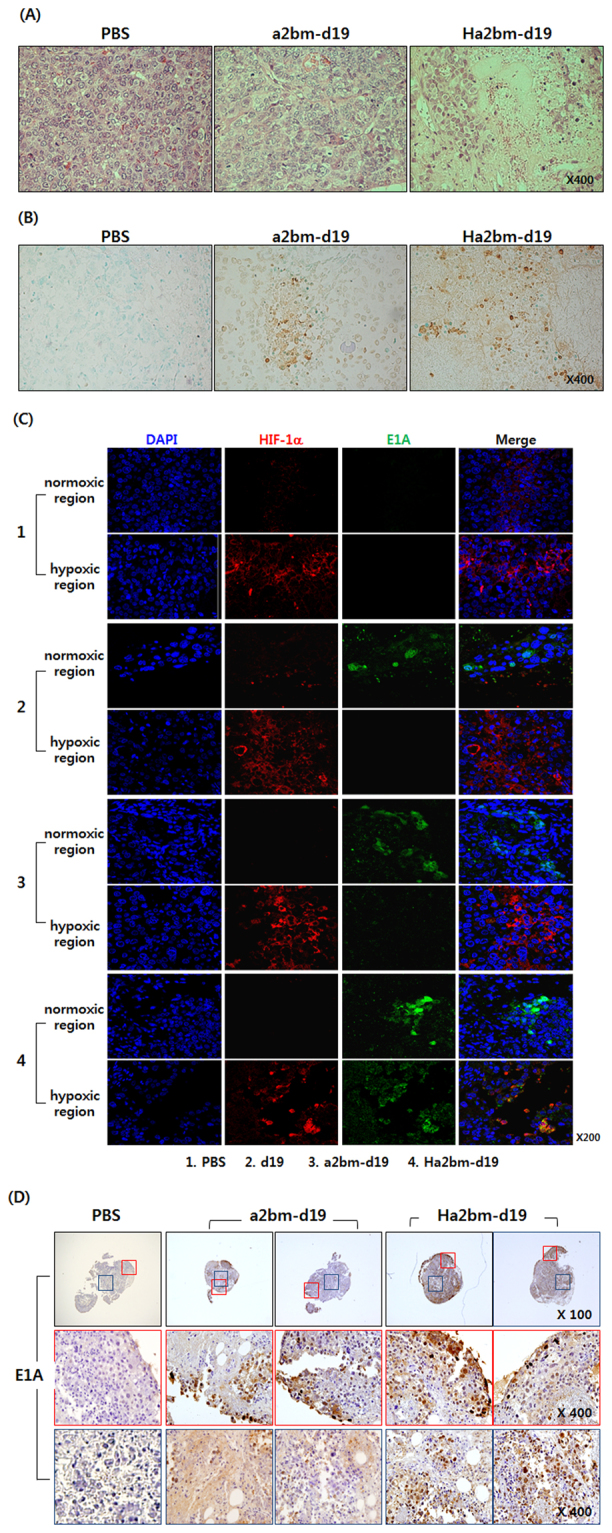
Histological and immunohistochemical changes in Hep3B xenograft tumors and spheroids treated with HCC-targeting oncolytic Ad. (**A**) Representative tumor sections were stained with hematoxylin and eosin (**H** & **E**). Data are presented at the original magnification of x400. (**B**) Apoptotic cells in Hep3B orthotopic tumor tissues were detected by terminal deoxynucleotidyl transferase dUTP nick end labeling (TUNEL) assay. Cells were counterstained with methyl green. Data are presented at the original magnification of x400 (**C**) Distribution of oncolytic Ads in tumor tissue were assessed by immunofluorescence staining using an Ad E1A-specific Ab. Hep3B orthotopic tumor sections were stained by an anti-Ad E1A Ab to examine virus replication (green). HIF-1α Ab was used to detect the hypoxic tumor regions (red). Sections were counterstained with DAPI (blue). Data are presented at the original magnification of x200. (**D**) Hep3B tumors were dissected into 1 mm^3^ organoids and then treated with 1 × 10^8^ VP of a2bm-d19 or Ha2bm-d19; the control was treated with PBS. Sections, containing tumor organoids, were stained with an anti-Ad E1A Ab (brown). Red boxes represent the expected normoxic (peripheral tumor) regions, whereas blue boxes indicate the expected hypoxic (central tumor) regions. Data are presented at the original magnification of x100 and x400.

To further assess the mechanism behind the potent antitumor effect of HCC-specific oncolytic Ads, tumor tissues were analyzed by histological examination. As shown in Fig. 5A, hematoxylin and eosin (H & E) staining revealed large areas of proliferating tumor cells in PBS-treated tissues, whereas a markedly reduced number of tumor cells, and moderate or extensive necrosis, were observed in a2bm-d19-, or Ha2bm-d19-treated tumor tissues, respectively. Further, Ha2bm-d19-treated tumor tissues exhibited more TUNEL-positive spots, which positively correlated with the degree of viral dispersion in tumor tissue, than did those treated with a2bm-d19 (Fig. 5B**)**. Of note, Ad E1A was detected in the normoxic regions of d19-, a2bm-d19-, or Ha2bm-d19-treated tumors, whereas only Ha2bm-d19 was detected in abundance within the hypoxic tumor region (HIF-1α-positive) with no spots being observed for d19- or a2bm-d19-treated tumors (Fig. 5C**)**. These results demonstrate that Ha2bm-d19 can overcome hypoxia-mediated downregulation of Ad replication and replicate effectively in both normoxic and hypoxic tumor regions. In line with these results, Ha2bm-d19-treated tumor spheroid showed an extensive distribution of viral progenies in both the peripheral and central tumor regions, whereas the virus was only observed in the peripheral regions of a2bm-d19-treated spheroids (Fig. 5D**)**. These results demonstrate that hypoxia-responsiveness of Ha2bm-d19 may facilitate viral replication in the central tumor region, which is known to be more hypoxic with high interstitial pressure because of inadequate vascularization^37,38^. We have also found that all these trends were reproducible in subcutaneous Hep3B tumor xenograft model (Fig. S1). Collectively, these results indicate that the hypoxia-responsive and enhancer region-modified Ha2bm promoter can greatly augment the replication of oncolytic Ads and leads to a potent oncolytic effect against HCC.

## Discussion

The enhancers and silencers, located upstream of promoters, interact with cell-specific transcriptional microenvironments and regulate gene expression^20–22^. The AFP promoter possesses several well-defined enhancers and silencers, in the regulatory region located far upstream, which regulate the expression of AFP^16,25,39^. Based on these reports, we inserted several different chimeric enhancer/silencer combinations upstream of the AFP promoter to induce optimal replication of oncolytic Ads in a HCC-specific manner, eliciting a potent antitumor response.

The initial screening of luciferase-expressing plasmid vectors revealed that the a2bm promoter, containing two E_A_ and one E_B_ regions upstream of the minimal AFP promoter, induced a significantly higher expression of luciferase than those expressed by other promoter variants (abm and a2bSm). Of note, a2bm promoter induced higher transgene expression than full length human AFP (hAFP) promoter (Fig. S2A). Further, Ha2bm promoter-driven oncolytic Ad elicited more potent reduction in viability of cancer cells than hypoxia-responsive full human AFP promoter-driven oncolytic Ad (HRE6-hAFP-d19^40^) in AFP-positive HCC cells (Fig. S2B), suggesting that deletion of the silencers and insertion of the extra E_A_ region to enhancer region containing single E_B_ can greatly augment the transcriptional activity of the AFP promoter (Fig. 1). These findings agree with previous reports showing that the effect of E_B_ on transcription is lower than that of E_A_, likely because E_A_ contains more binding sites for the liver-enriched transcription factors, which are overexpressed in HCCs^41,42^. Silencers prevent promoter activation in non-target cells and endow the promoter with specificity^43,44^. The deletion of endogenous silencer domains did not affect the cancer specificity of the AFP promoter variants, showing minimal promoter activity in normal cells (Fig. 2). Additionally, the removal of silencers greatly enhanced the transcriptional activity of the AFP promoter in AFP-positive cancer cells. Together, these results suggest that the silencer domains of the AFP promoter are not critical for efficient targeting of HCCs.

Several strategies, such as addition, deletion, or chimerization of promoter domains, have been used to enable promoters to exploit constitutively active cancer signaling pathways or transcription factors overexpressed in the tumor microenvironment^45–47^. Among several candidates, HREs are frequently utilized, because they can be activated by numerous types of tumors from different tissue origins, and because hypoxia is a common attribute of solid tumors^40^. As shown in Fig. 2, the transcriptional activity of the hypoxia-responsive promoter variants Ha2bm and Ha2bSm was markedly higher than those of the control promoters a2bm and a2bSm, respectively, under hypoxia. These results agree with other reports demonstrating that HREs can augment the promoter activity under hypoxic conditions^40,48,49^. This responsiveness under hypoxic conditions was cancer-specific because the Ha2bm and Ha2bSm promoters retained their cancer specificity and remained inactive in normal cells (HEP-187M) under normoxia and hypoxia. Similarly, the Ha2bm promoter-regulated oncolytic Ad showed more potent reduction in viability of cancer cells than did the a2bm promoter-driven oncolytic Ads in AFP-positive and -negative HCCs (Fig. 3B). These results demonstrate that the Ha2bm promoter-driven oncolytic Ads can target hepatoma cells regardless of their expression levels of AFP, which is strongly implicated in 30% of the patients, diagnosed with HCC, who show normal levels of AFP^50^.

Consistent with these *in vitro* results, the oncolytic Ad, replicating under the control of the Ha2bm promoter (Ha2bm-d19), showed higher HCC-specific and potent antitumor activity than did the a2bm-d19-driven Ad in AFP-positive HCC orthotopic xenograft tumors; this is because HREs, upstream of the Ha2bm promoter, enhance viral replication and gene expression in the hypoxic tumor region^40,51–53^ (Figs 2C, 4E and 5C). The Ha2bm promoter enabled the oncolytic Ad to actively replicate and disperse through the central and hypoxic tumor regions, expressing high levels of HIF-1α. This shows that Ha2bm-d19 can overcome the hypoxia-mediated downregulation of Ad replication in solid tumors^37,38^, resulting in a potent induction of tumor cell apoptosis^37,38^ (Fig. 5C and D**)**. In support of these findings, Ha2bm-d19-treated mice exhibited the lowest increase in the serum levels of AFP, which is a good biomarker for the determination of disease progression and malignancy in AFP-positive HCCs^33,54,55^^,^. These results suggest that Ha2bm-d19 may effectively suppress the proliferation and angiogenesis, associated with high levels of AFP, in a clinical setting^56^.

Following systemic administration of the oncolytic Ads, Ha2bm-d19-treated mice showed the highest quantity of virion in tumor tissues likely due to superior replication of Ha2bm-d19 in hypoxic tumor region (Figs 4E and 5C). These findings are in line with our *in vitro* results where Ha2bm-d19 showed 63.1-fold higher viral replication than a2bm-d19 under hypoxic conditions (Fig. S3). Importantly, both a2bm-d19- and Ha2bm-d19-treated mice induced a markedly lower level of the innate immune response and hepatotoxicity compared with those induced by the replication-competent Ad lacking cancer specificity (d19) (Fig. 4F and G), further reaffirming that small quantity of virions delivered to normal hepatic tissue did not replicate as efficiently as it did in tumor tissues to induce observable cytopathic effect (Fig. 4E). This demonstrates that HCC specificity, endowed by the Ha2bm promoter, can attenuate the undesirable side effects of systemically administered Ads. This result agrees with the report in which AFP was locally overexpressed in the tumor microenvironment and expressed at a low level in the surrounding normal hepatic tissues^21^. These attributes are important for the successful treatment of clinical tumors because most intestinal tumors and distant metastases are difficult to treat by local administration^57^. In summary, systemic administration of the oncolytic Ad, replicating under the control of tumor microenvironment- and HCC-targeting AFP promoter, can induce a potent inhibition of tumor growth, with a good safety profile, rendering it a promising candidate for the treatment of primary tumors and distant metastases in patients with late stages of cancer.

## Materials and Methods

### Cell lines and cell culture

HEK293 (human embryonic kidney cell line expressing the adenoviral E1 region), AFP-positive HCC (Huh7, HepG2, and Hep3B), AFP-negative HCC (HepI), brain cancer cell line (U343), non-small lung cancer cell line (A549), and human fibroblast cell lines (BJ, WI38, and IMR90) were purchased from the American Type Culture Collection (ATCC, Manassas, VA). These cell lines were cultured in Dulbecco’s Modified Eagle’s Medium (Gibco BRL, Gaithersburg, MD) or Modified Eagle’s Medium (Gibco BRL) supplemented with 10% fetal bovine serum (Gibco BRL), penicillin (100 U/mL), and streptomycin (50 mg/mL). Primary human hepatocyte (HEP-187M) was purchased from the Biopredic International (Rennes, France), and cultured in basal hepatic cell medium (Buckingham, UK) supplemented with additives for hepatocytes incubation medium (Buckingham). All cell lines were maintained at 37 °C in a humidified incubator at 5% CO_2_. Hypoxia (oxygen concentration of 1%) was achieved using pre-warmed aluminum hypoxic chambers evacuated.

### Construction of vectors controlled by modified AFP promoter

To generate several AFP promoter variants with a reconstituted enhancer domain in the upstream region, E_A_ (365 bp) and E_B_ (192 bp) enhancer segments, located within -4.12 to -3.76 kb and -3.49 to -3.30 kb regions of the full length enhancer, respectively, were isolated from phAFP5.1 (provided by T. Tamaoki, University of Calgary, Canada) and amplified by PCR^3,11^. Similarly, the position-dependent distal silencer (S_d_; 90 bp) and proximal silencer (S_P_; 156 bp), located between the −3.01 to −1.75 kb region, were isolated and amplified by PCR. The full length enhancer (Ef; 1150 bp) was also amplified from phAFP5.1 by PCR. Subsequently, various combinations of these enhancer and silencer PCR constructs were inserted upstream of the minimal AFP promoter, generating abm, a2bm, a2bSm, and EfSm promoters containing varying copy numbers of E_A_, E_B_, S_d_, S_p_, and Ef (Fig. 1A). To assess the transcriptional activity of these promoters, the endogenous CMV promoter of luciferase-expressing pGL3 basic vector (denoted as pGL3-CMV; Promega, Madison, WI) was replaced with promoter variants, generating pGL3-AFPm, pGL3-abm, pGL3-a2bm, pGL3-a2bSm, and pGL3-EfSm. Replication-incompetent Ads expressing the green fluorescence protein (GFP) under the control of CMV, AFPm, a2bm, and a2bSm promoters were generated, resulting in dAd/CMV-GFP, dAd/AFPm-GFP, dAd/a2bm-GFP, and dAd/a2bSm-GFP, respectively. Next, a2bm and a2bSm promoters, containing six copies of HRE in the upstream region (Ha2bm and Ha2bSm, respectively), were constructed to drive the expression of GFP, generating dAd/Ha2bm-GFP and dAd/Ha2bSm-GFP, respectively. Subsequently, HCC-specific oncolytic Ads were generated by replacing the endogenous Ad E1A promoter of the replication-competent Ad d19^34^ with the a2bm or Ha2bm promoter, generating a2bm-d19 and Ha2bm-d19. Propagation, purification, and titration of all the Ads, used in this study, were performed as previously described^58^. The number of viral particles (VP) was calculated from an optical density measurement at 260 nm (OD_260_), where the absorbance of 1 (OD_260_ = 1) was equivalent to 1.1 × 10^12^ VP/mL. Purified viruses were stored at −80 °C until use.

### Enzyme-linked immunosorbent assay

To investigate the AFP expression level of each cell lines, 1 × 10^5^ cells were seeded onto 6-well plates for 48 h. AFP concentration was then measured in culture supernatants by ELISA according to instructions provided by the vendor (R&D Systems, Minneapolis, MN).

### Luciferase activity assays

Luciferase activity assays were performed using pGL3/CMV, pGL3/AFPm, pGL3/abm, pGL3/a2bm, pGL3/a2bSm, and pGL3/EfSm reporter plasmids in several cancer cell lines [(AFP-positive HCC cells: Huh7, HepG2, and Hep3B, AFP-negative HCC cells: HepI, non-HCC cancer cells: U343 and A549)] and normal cells (fibroblasts: BJ, WI38, CBHEL, and IMR90, primary human hepatocytes: HEP-187M). Luciferase activity was measured, as described in the manufacturer’s manual for the Luciferase assay kit (Promega), using a luminometer and normalized to the total protein concentration. Total protein concentration was measured with a BCA protein assay kit (Pierce Biotechnology, Rockford, IL). Luciferase activity was expressed as total units of luciferase per total protein content. Experiments were carried out in triplicate and repeated at least three times.

### Fluorescence-activated cell-sorting analysis

The fluorescence-activated cell sorting (FACS) analysis was conducted to assess the specificity and transcriptional activity of modified AFP promoters with HREs located upstream. Cells were transduced with dAd/CMV-GFP, dAd/AFPm-GFP, dAd/a2bm-GFP, dAd/a2bSm-GFP, dAd/Ha2bm-GFP, or dAd/Ha2bSm-GFP (Huh7 and A549 at 40 MOI, HepG2 at 30 MOI, Hep3B and Hep1 at 20 MOI, and BJ at 100 MOI). At 48 h post transduction, cells were harvested using a dissociation solution (Sigma, St. Louis, MO) and washed with phosphate buffered saline (PBS). GFP expression levels from each group were analyzed by FACScan (Beckton-Dickinson, East Rutherford, NJ). Data were collected from 10,000 cells and analyzed with the CellQuest software (BD Biosciences Immunocytometry Systems, San Jose, CA).

To compare the promoter strength of full length hAFP promoter and a2bm promoter, Hep3B and Huh7 cells were transduced with replication-incompetent Ad expressing GFP under the control of hAFP promoter (dAd/hAFP-GFP) or dAd/a2bm-GFP at 40 or 50 MOI, respectively. GFP expression levels from each group were analyzed by FACScan (Beckton-Dickinson) as described above.

### MTT assay

To assess the HCC-specific killing effect of the oncolytic Ads, controlled by the modified AFP promoter, cells were seeded onto 24-well plates at 2 × 10^4^ cells per well. Cells were infected with d19, a2bm-d19, or Ha2bm-d19 and incubated under hypoxia or normoxia at 37 °C. At 3 to 5 days post infection, 200 μL of 3-(4,5-dimethylthiazolyl-2-yl)-2,5-diphenyltetrazolium bromide (MTT; Sigma) in PBS (2 mg/mL) was added to each well following removal of the media. Cells were then incubated for 4 h at 37 °C in a humidified incubator at 5% CO_2_. The solution was removed, and formazan crystals were dissolved with 1 mL dimethylsulfoxide (DMSO). Cell viability was determined by measuring the absorbance at 540 nm using a microplate reader (Bio-RAD, Hercules, CA). Untreated groups for each day were designated as 100% viable.

To compare the potency of enhancer region modified and hypoxia-responsive Ha2bm-driven oncolytic Ad with our previous hypoxia-responsive HCC-specific promoter-driven oncolytic Ad (HRE6-hAFP-d19), Hep3B cells were infected with HRE6-hAFP-d19 or Ha2bm-d19 for 24 h and then MTT assay was performed as described above.

### Virus production assay

To assess the viral production of oncolytic Ads, Hep3B were seeded in 24 well plates and then infected with a2bm or Ha2bm at an MOI of 0.2. After 2 days of incubation at 37 °C under hypoxic conditions, supernatants and cell pellets were collected and freeze-thawed 3 times to harvest both cellular and extracellular virions. Real-time quantitative PCR (Q-PCR; TaqMan PCR detection; Applied Biosystems, Foster City, CA) was used to assess the number of viral genomes in each sample as described previously^57^. Samples were analyzed in triplicates and data were processed using the SDS 19.1 software package (Applied Biosystems).

### Assessment of antitumor effect in an orthotopic hepatoma model

Hep3B cells (1 × 10^6^), which stably express the firefly luciferase gene, were injected into the left liver lobe of athymic nude mice. At 14 days post implantation, blood was harvested by retro-orbital bleeding, and the level of AFP was analyzed by enzyme-linked immunosorbent assay (ELISA) (R&D Systems, Minneapolis, MN) according to manufacturer’s instruction. At 14 days post implantation, the serum level of AFP was analyzed every day by retro-orbital bleeding until it reached 300 ng/mL. When the level of AFP expression reached approximately 300 ng/mL, the mice were randomly divided into four groups and treated with an intravenous injection of PBS, or 2.5 × 10^10^ VP of d19, a2bm-d19, or Ha2bm-d19 every 2 days for a total of three times (n = 6 per group). Optical imaging, with an IVIS SPECTRUM instrument (Xenogen Corp., Alameda, CA), was conducted every week following the first treatment. Image signals were quantitatively analyzed with IGOR-PRO Living Image software (Xenogen). Blood was collected at week 5 to assess the final serum levels of AFP using ELISA as described above in section 2.6. The percentage of surviving mice was determined by monitoring the tumor growth-related events (ROI (p/s) > 2 × 10^9^ for orthotopic tumor) over a period of 5 weeks.

### Biodistribution assessment by real-time quantitative PCR

Once the Hep3B orthotopic tumor were established, tumor-bearing mice were injected intravenously with 2.5 × 10^10^ VP of a2bm-d19 or Ha2bm-d19 three times every other day along with PBS as a negative control. The blood, muscle, lung, heart, kidney, spleen, liver, and tumor tissues were harvested at 72 h after the third injection of each virus, and DNA was extracted from the tissues using the QIAamp DNA Blood Mini Kit (Qiagen) according to the manufacturer’s instructions^57^. The number of Ad genomes was measured by Q-PCR (Applied Biosystems). Samples were analyzed in triplicates and data were processed by the SDS 19.1 software package (Applied Biosystems).

### Histology and immunohistochemistry

To establish Hep3B tumors, 1 × 10^7^ cells of Hep3B were injected subcutaneously into the abdomen of 6-week-old male athymic nu/nu mice. When the tumor volume reached approximately 150 to 200 mm^3^, each animal received intratumoral injections of PBS, dAd/a2bm-GFP, or dAd/Ha2bm-GFP (5 × 10^9^ VP) every 2 days for a total of three times. Seven days after the final injection, tumor tissues were treated with pimonidazole (Sigma). Tumors were collected and fixed in 10% formalin and embedded in paraffin. Tumor sections were stained with an anti-GFP monoclonal antibody (Ab) (Chemicon, Temecula, CA) and pimonidazole hydrochloride (hypoxyprobe-1, Chemicon). The slides were mounted with Vectashield mounting medium (Vector Laboratories, Burlingame, CA) and tissues were viewed under a confocal laser-scanning microscope (LSM510, Carl Zeiss MicroImaging, Thornwood, NY).

To evaluate the antitumor efficacy of HCC-targeted oncolytic Ads, mice were treated with an intravenous injection of PBS, a2bm-d19, or Ha2bm-d19 (2.5 × 10^10^ VP) every 2 days for a total of three times. Tumor tissues were collected 7 days post final injection. Representative sections were stained with hematoxylin (Sigma, Munich, Germany) and eosin (Sigma, Munich, Germany) (H & E). Apoptosis in tumor tissue was detected by terminal deoxynucleotidyl transferase dUTP nick end labeling (TUNEL) assay (DeadEndTM Fluorometric TUNEL System; Promega)^59^. Briefly, tissue sections were permeabilized with proteinase K (20 mg/mL) for 10 min at room temperature. Sections were then incubated with terminal deoxynucleotidyl transferase (TdT) and fluorescein-12-dUTP in TdT buffer at room temperature for 60 min and washed with TdT buffer. Finally, nuclei were counterstained with methyl green (Sigma). The samples were analyzed by light microscopy (Carl Zeiss MicroImaging).

### Immunofluorescence staining

To evaluate the antitumor efficacy of HCC-targeted oncolytic Ads, Hep3B HCC-bearing mice were treated by intravenous injection with PBS, d19, a2bm-d19, or Ha2bm-d19 (2.5 × 10^10^ VP) every 2 days for a total of three times. Tumor tissues were collected 7 days post final injection. For immunofluorescence staining of Ad E1A and HIF-1α in tumor tissues, tumor sections were treated with rabbit anti-Ad E1A Ab (Chemicon) or mouse anti-human HIF-1α Ab (Abcam, Cambridge UK) and incubated overnight at 4 °C. Next, the tumor sections were incubated with Alexa Fluor 488 (green)-conjugated goat anti-rabbit IgG (Invitrogen) or Alexa Fluor 568 (red)-conjugated goat anti-mouse IgG (Invitrogen) at room temperature for 1 h. The slides were mounted with Vectashield mounting medium (Vector Laboratories) and imaged under a confocal laser-scanning microscope (LSM510, Carl Zeiss MicroImaging, Thornwood, NY).

### Organoid culture and immunohistochemistry

Tumor organoid culture was established by dissecting subcutaneously established Hep3B tumors into 1-mm diameter pieces using sterile 21-gauge needles. The explants were plated onto HydroCell^®^ 24 multi-well plates (Nunc, Rochester, NY) and cultured for 4 h in Iscove’s Modified Dulbecco’s Medium (GIBCO) supplemented with 5% FBS, 10 mM insulin (Sigma), and 1 mM hydrocortisone (Sigma). HCC organoids were subsequently treated with 1 × 10^8^ VP of a2bm-d19 or Ha2bm-d19, or with PBS as control, and incubated at 37 °C for 3 days. To evaluate viral replication in tumor organoids, representative organoid tissue sections were incubated at 4 °C overnight with rabbit anti-Ad E1A primary Ab (Chemicon) and then processed with the ABC-peroxidase kit (Thermo Fisher Scientific).

### Ethics Statement

All facilities were approved by the Association for Assessment and Accreditation of Laboratory Animal Care. All animal experiments were conducted according to the institutional guidelines established by the Hanyang University Institutional Animal Care and Use Committee.

### Statistical analysis

Data were expressed as the mean ± standard deviation (SD). Statistical significance was determined by two-tailed Student’s t-test or one-way ANOVA (SPSS 13.0 software; SPSS, Chicago, IL). *P* values less than 0.05 were considered statistically significant.

## Electronic supplementary material

Supplementary information
