# Supplementary material for: Hepatocellular carcinoma-targeting oncolytic adenovirus overcomes hypoxic tumor microenvironment and effectively disperses through both central and peripheral tumor regions
Source: Sci Rep. 2018 Feb 2;8:2233. doi: 10.1038/s41598-018-20268-6 (PMC5797125; doi:10.1038/s41598-018-20268-6)
Supplement: Supplementary file 1 — Supplementary information [file 41598_2018_20268_MOESM1_ESM.pdf]

Hepatocellular carcinoma-targeting oncolytic adenovirus overcomes hypoxic tumor microenvironment and effectively disperses through both central and peripheral tumor regions

A-Rum Yoon[1], JinWoo Hong[1], Minjung Kim[1], Chae-Ok Yun[1*]

[1]Department of Bioengineering, College of Engineering, Hanyang University

222 Wangsimni-ro, Seongdong-gu, Seoul 04763, Korea

Fig. S1

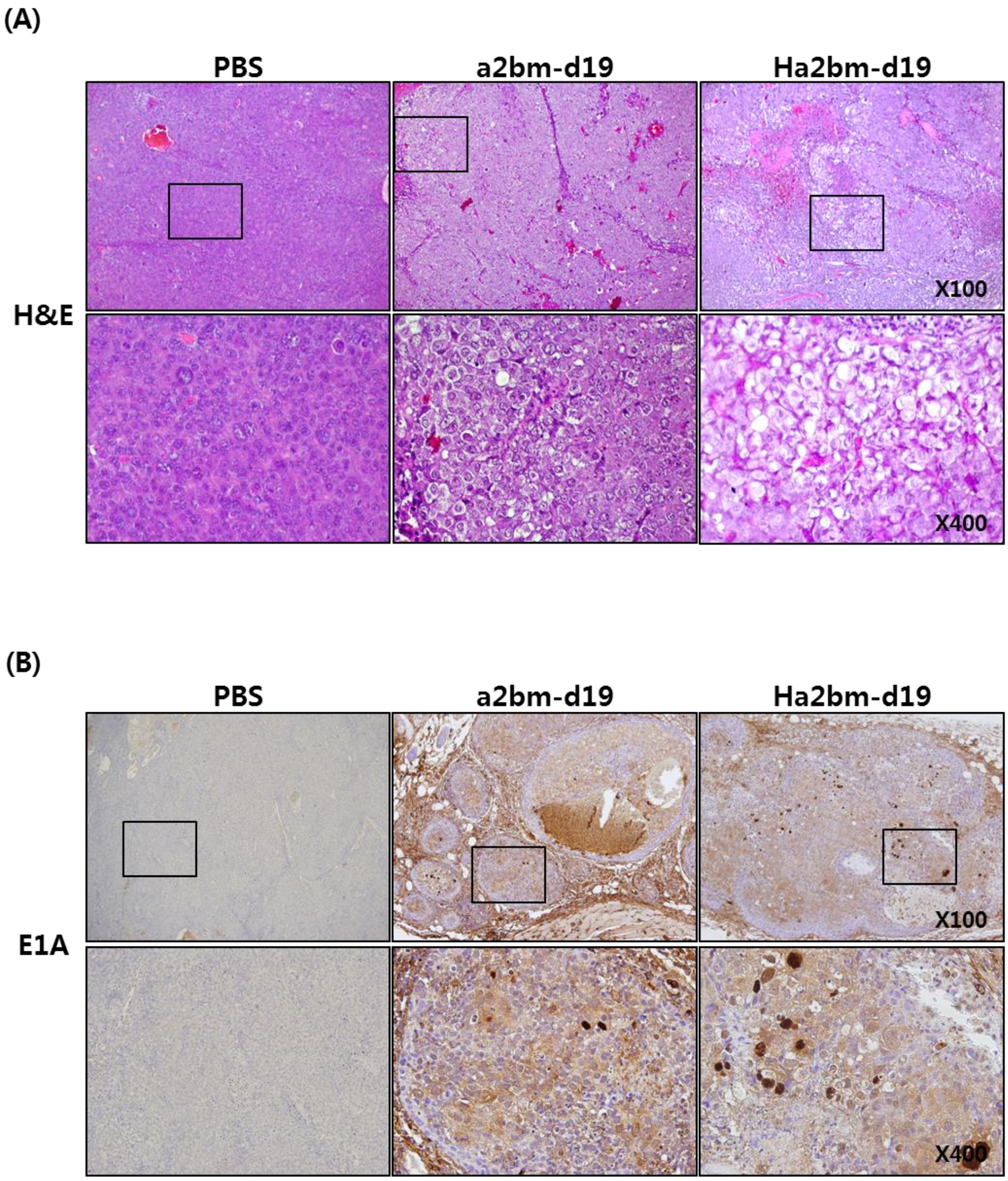
(A)
PBS
a2bm-d19
Ha2bm-d19
H&E
X100
X400
(B)
PBS
a2bm-d19
Ha2bm-d19
E1A
X100
X400

(C)

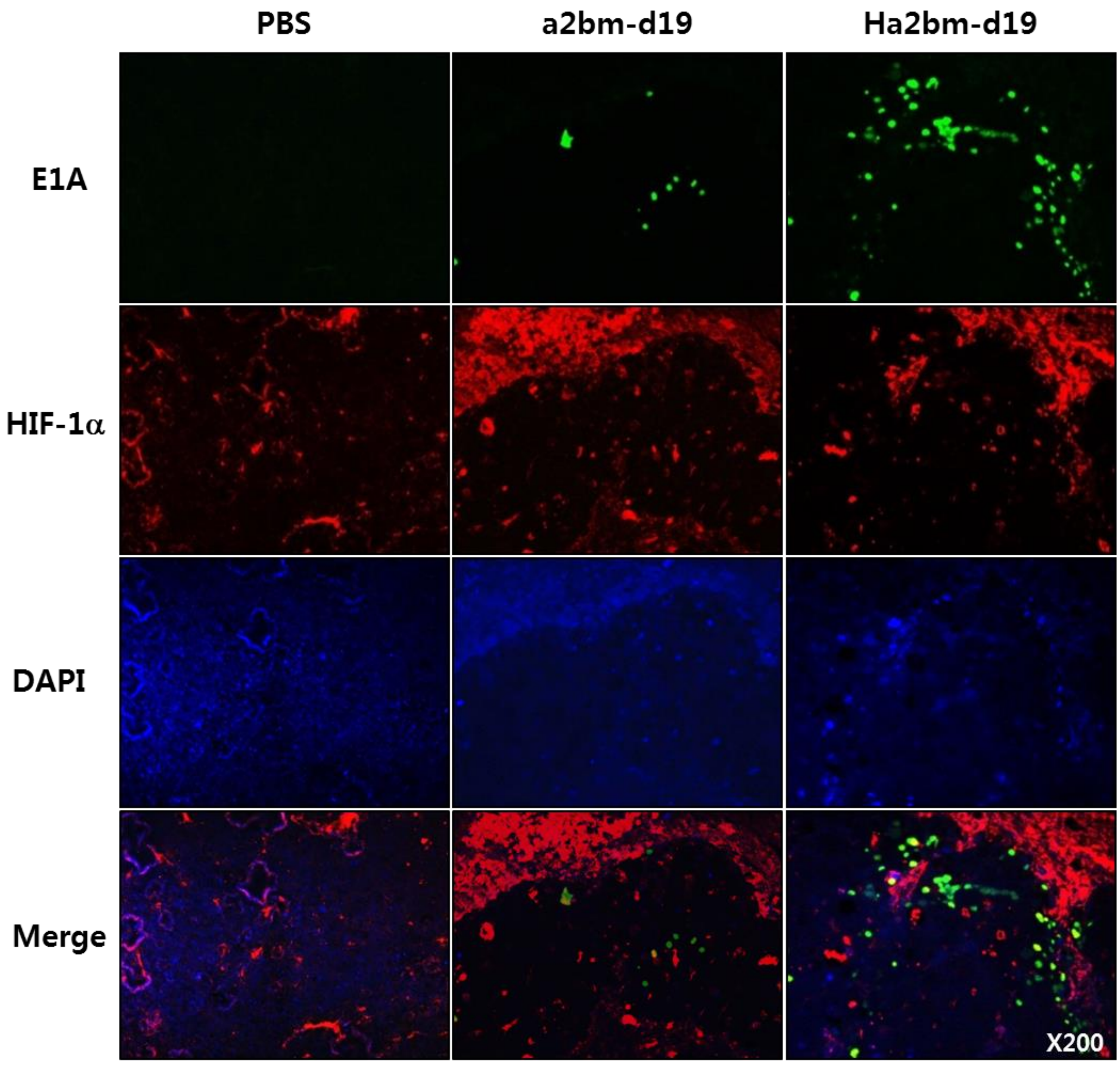

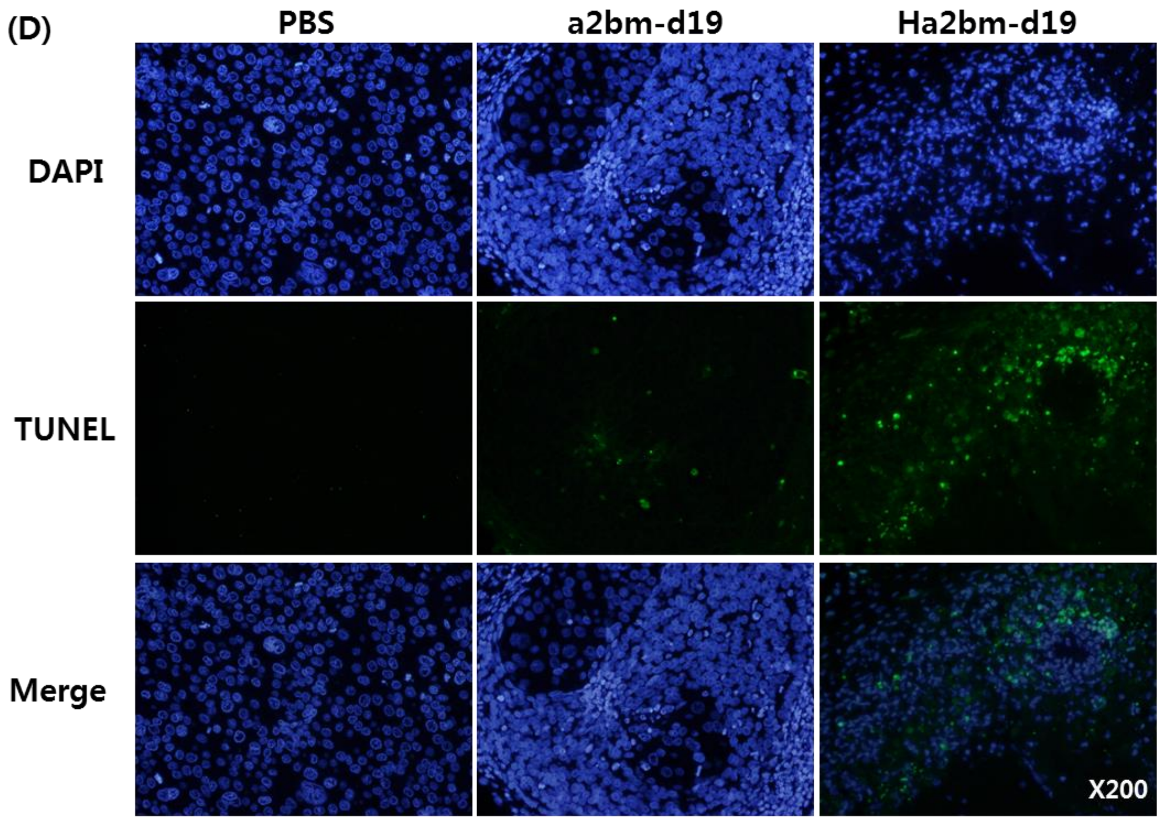

Supplementary Figure S1. Histological and immunohistochemical changes in subcutaneous Hep3B xenograft tumors and spheroids treated with HCC-targeting oncolytic Ad. (A) Representative tumor sections were stained with hematoxylin and eosin (H & E). Data are presented at the original magnification of x100 and x400. (B) Intratumoral distribution of oncolytic Ads was assessed by immunohistochemistry using an Ad E1A-specific Ab. Data are presented at the original magnification of x100 and x400. (C) Hep3B tumor sections were stained by an anti-Ad E1A Ab to examine virus replication (green). HIF-1α Ab was used to detect the hypoxic tumor regions (red). Sections were counterstained with DAPI (blue). Data are presented at the original magnification of x200. (D) Apoptotic cells in Hep3B tumor tissues were detected by terminal deoxynucleotidyl transferase dUTP nick end labeling (TUNEL) assay. Cells were counterstained with DAPI (blue). Data are presented at the original magnification of x200.

Fig. S2

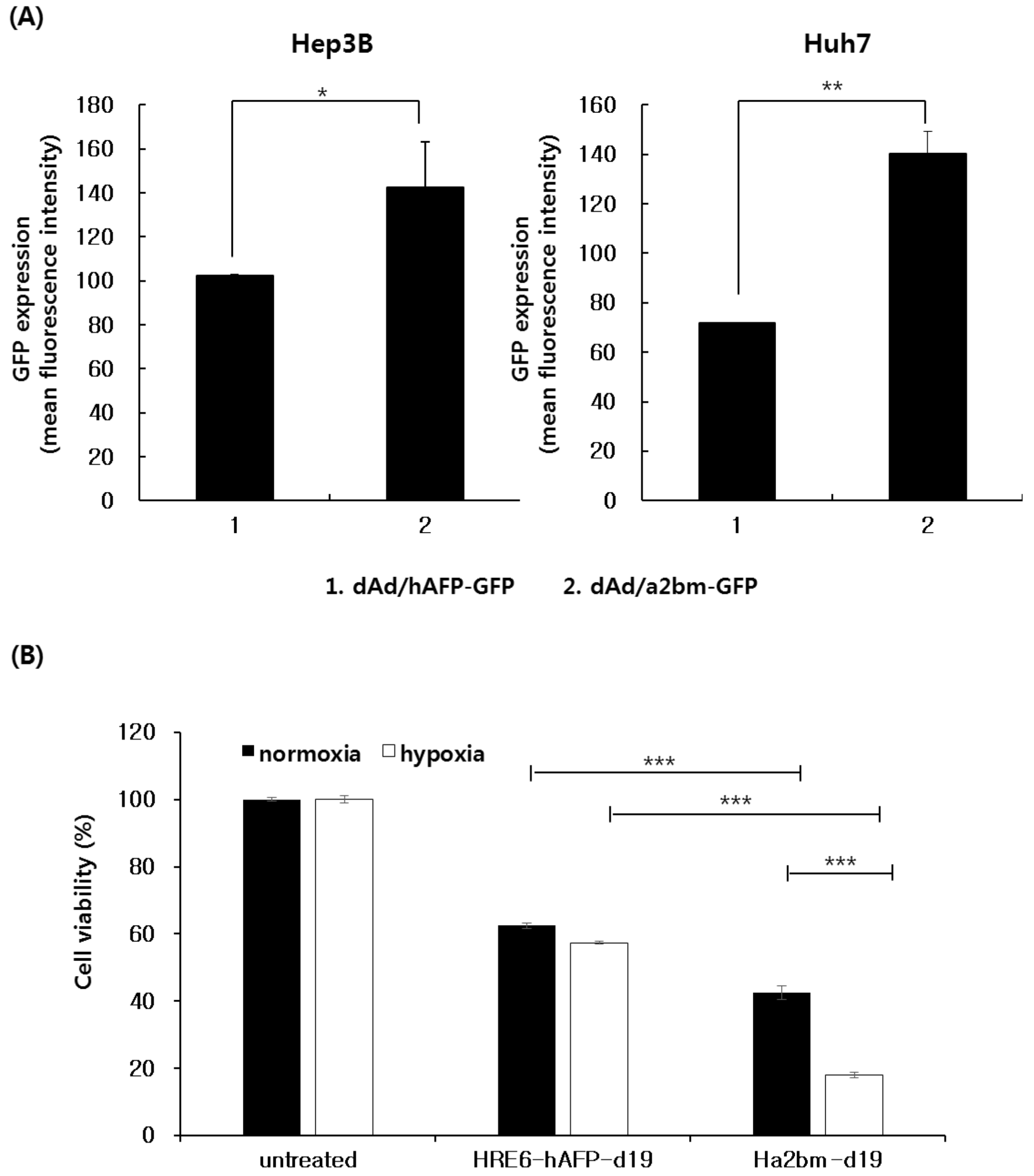

Supplementary Figure S2. Comparison of transgene expression mediated by replication-incompetent Ads expressing GFP under the control of full length human AFP promoter or chimeric a2bm promoter. (A) AFP-positive HCC cells were transduced with dAd/hAFP-GFP or dAd/a2bSm-GFP (Hep3B; 40 MOI and Huh7; 50 MOI). The expression levels of GFP

were analyzed at 48 h post transduction by FACS. The expression of GFP was normalized to the expression of GFP mediated by PBS-treated group. Each cell line was tested at least three times and the data shown are representative of experiments performed in triplicate. Bars represent mean ± SD. *$P < 0.05$, **$P < 0.01$. (B) Hep3B cells were infected with HRE6-hAFP-d19 or Ha2bm-d19 for 24 h and then cell viability was assessed by MTT assay. Bars represent mean ± SD. ***$P < 0.001$

Fig. S3

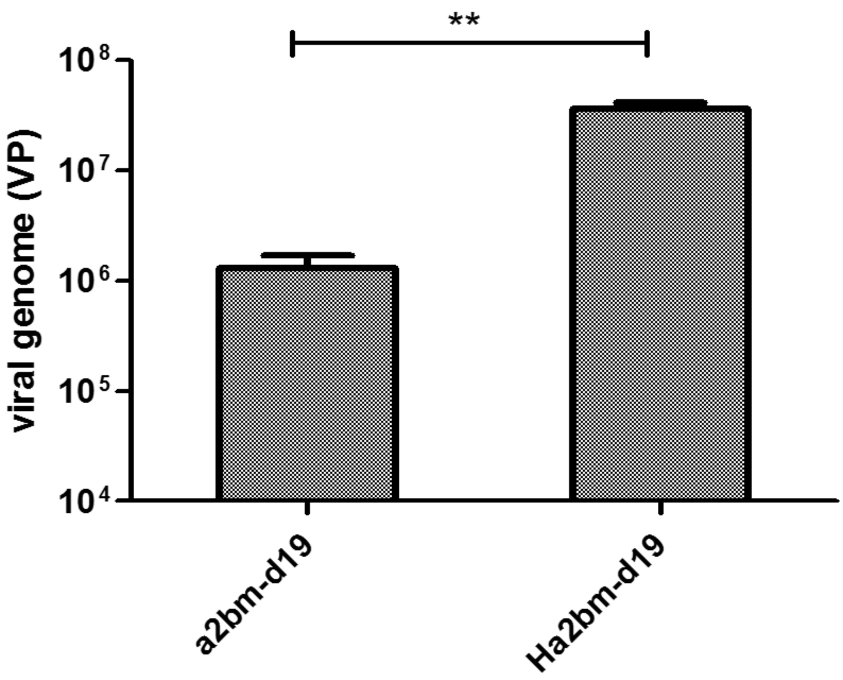

Supplementary Figure S3. Viral production of HCC-targeting oncolytic Ads under hypoxia. Hep3B cells were infected with a2bm-d19 or Ha2bm-d19 at 0.2 MOI under hypoxic condition. 2 days post infection, supernatant and cell lysates were harvested and these samples underwent 3 cycles of freeze-thawing. Real-time quantitative PCR was used to assess the number of viral genomes in each sample. The experiments were repeated at least three times and the data shown are representative of experiments performed in triplicate. Bars represent mean ± SD. $^{**}P < 0.01$.

Supplementary Table 1. Raw data of luciferase assay in various cell lines

Luciferase expression (RLU/µg)	**CMV**	**AFPm**	**abm**	**a2bm**	**a2bSm**	**EfSm**
**Huh7**	6776530.0 ± 687445.3	1984.6 ± 314.1	716215.6 ± 65813.3	1998376.3 ±187518.2	942512.3 ± 55859.7	902865.3 ± 11607.3
**HepG2**	728595.0 ± 15216.3	913.9 ± 153.5	334593.3 ± 25801.6	608553.3 ± 22817.5	178314.0 ± 22817.5	96029.0 ± 178.4
**Hep3B**	1005732.4 ± 28946.8	1655.4 ± 2.5	111135.0 ± 370.8	258624.0 ± 3960.6	140080.4 ± 8426.0	236612.7 ± 9839.8
**HepI**	444336.0 ± 13405.3	137.4 ± 21.0	13.7 ± 8.7	74.7 ± 15.6	56.7 ± 19.2	143.7 ± 5.3
**U343**	133154.7 ± 15269.7	7.3 ± 1.5	133.7± 134.6	130.7 ± 170.1	87.7 ± 71.8	48.3 ± 5.6
**A549**	720198.0 ± 109707.1	112.7 ± 20.5	2117.3 ± 568.7	1861.0 ± 507.2	417.7 ± 93.8	265.0 ± 57.5
**BJ**	6661.3 ± 35.7	2.0 ± 2.6	4.0 ± 6.0	6.0 ± 1.0	10.3 ± 1.5	7.3 ± 1.5
**WI38**	23932.7 ± 10990.2	2.4 ± 7.6	70.7 ± 19.1	11.0 ± 10.1	8.4 ± 9.5	23.7 ± 13.1
**IMR90**	26322.3 ± 9822.4	5.0 ± 3.1	31.6 ± 27.0	22.3 ± 10.6	20.3 ± 10.4	10.6 ± 7.1

**HEP-187M**	21989.0 ± 3022.3	21.3 ± 4.0	40.7 ± 9.0	18.0 ± 8.7	112.7 ± 173.5	18.7 ± 6.5

Supplementary Table 2. Raw data of FACS assessing GFP expression in various cell lines (Mean value)

Mean Value	dAd/CMV -GFP		dAd/AFPm -GFP		dAd/a2bm -GFP		dAd/a2bSm -GFP		dAd/Ha2bm -GFP		dAd/Ha2bSm -GFP	
	**N**	**H**	**N**	**H**	**N**	**H**	**N**	**H**	**N**	**H**	**N**	**H**
**Huh7**	**2066 ± 52**	**2356 ± 101**	**50 ± 12**	**70 ± 5**	**416 ± 32**	**478 ± 50**	**341 ± 69**	**435 ± 14**	**851 ± 29**	**1818 ± 282**	**575 ± 67**	**1393 ± 166**
**HepG2**	**1156 ± 24**	**833 ± 114**	**31 ± 7**	**31 ± 2**	**145 ± 5**	**150 ± 34**	**84 ± 8**	**95 ± 7**	**157 ± 19**	**774 ± 79**	**83 ± 13**	**354 ± 32**
**Hep3B**	**81 ± 3**	**51 ± 4**	**5. ± 1**	**4 ± 1**	**41 ± 0**	**2 ± 0**	**3 ± 1**	**2 ± 0**	**6 ± 0**	**57 ± 10**	**4 ± 1**	**26 ± 6**
**HepI**	**886 ± 111**	**889 ± 111**	**119 ± 7**	**121 ± 5**	**110 ± 16**	**112 ± 16**	**161 ± 17**	**163 ± 17**	**559 ± 23**	**561 ± 23**	**537 ± 44**	**529 ± 44**
**A549**	**559 ± 20**	**552 ± 25**	**23 ± 2**	**30 ± 5**	**11 ± 2**	**25 ± 1**	**10 ± 1**	**30 ± 3**	**22 ± 1**	**219 ± 2**	**14 ± 0**	**162 ± 1**
**BJ**	**11 ±0**	**12 ±0**	**1 ± 0**	**1 ± 0**	**1 ± 0**	**1 ± 0**	**1 ± 0**	**1 ± 0**	**4 ±0**	**3 ± 0**	**2 ± 0**	**2 ± 0**

N: normoxia, H: hypoxia
